# Risk Communication and Factors Influencing Private Well Testing Behavior: A Systematic Scoping Review

**DOI:** 10.3390/ijerph16224333

**Published:** 2019-11-07

**Authors:** Sarah K. Colley, Peter K.M. Kane, Jacqueline MacDonald Gibson

**Affiliations:** 1 Department of Environmental Sciences and Engineering, Gillings School of Global Public Health, University of North Carolina, Chapel Hill, NC 27599, USA; peterkanenc@gmail.com; 2 Department of Environmental and Occupational Health, School of Public Health, Indiana University, Bloomington, IN 47405, USA; jacmgibs@iu.edu

**Keywords:** private wells, well testing, drinking water, risk communication, health behavior, behavioral intervention

## Abstract

Unregulated private wells may be at risk for certain types of contamination associated with adverse health effects. Well water testing is a primary method to identify such risks, although testing rates are generally low. Risk communication is used as an intervention to promote private well testing behavior; however, little is known about whether these efforts are effective as well as the mechanisms that influence effectiveness. A systematic scoping review was conducted to evaluate the current evidence base for risk communication effectiveness and factors that influence well testing behavior. The review was conducted with the Preferred Reporting Items for Systematic Reviews and Meta-Analyses Extension for Scoping Reviews (PRISMA-ScR) framework. Data were synthesized using a health behavior model (Health Belief Model) to identify areas amenable to intervention and factors to consider when designing risk communication interventions. We identified a significant shortage of studies examining the effectiveness of risk communication interventions targeted to well testing behavior, with only two quasi-experimental studies identified. The review also identified seventeen studies that examined or described factors relating to well testing behavior. The two empirical studies suggest risk communication methods can be successful in motivating private well owners to test their water, while the remaining studies present considerations for developing effective, community-specific content.

## 1. Introduction

An estimated 42 million Americans and three million Canadians rely on unregulated private wells as their primary water supply [1,2]. While many private wells provide water of good quality, private wells may be at risk for certain types of contamination that are harmful to human health. Well water contaminants can include heavy metals, pathogenic microorganisms, nitrates, pesticides, motor vehicle fuel, and industrial chemicals, among others [3,4]. Many common well water contaminants are associated with undesirable health effects including neurological disorders, childhood development delays, endocrine and reproductive disruption, gastrointestinal illness, respiratory infections, and cancer [3,5,6,7]. The U.S. Geological Survey found that 23% percent of sampled private wells had one or more contaminants in exceedance of human-health standards [5]. Private well owners are responsible for ensuring that their water supply is safe and for performing well stewardship behaviors such as testing, maintenance, and treatment. Well water testing is the primary method by which well owners can identify potential contaminants in their drinking water. Testing is a critical first step in the sequence of events needed to mitigate hazards posed by unregulated private well water; test results—if appropriately conveyed, interpreted, and acted upon—can inform subsequent protective behaviors like filtration or treatment. Although suggested testing frequencies and analytes may vary by location, many federal, state, and provincial agencies recommend annual to biannual testing for most private well owners. Some estimates suggest less than one-third of private well owners adhere to this recommended testing schedule, and that less than half have tested their wells within the last decade [8,9,10,11,12]. Taken together, such data indicate that millions of North Americans regularly use water of either unknown quality or poor quality. The risks associated with low testing rates of private wells pose a preventable public health problem and an area for intervention.

To decrease these risks, external stakeholders such as local public health departments, universities, and cooperative extension programs often assume responsibility for educating and supporting private well owners [13,14,15]. Such external stakeholders may employ risk communication techniques to encourage private well testing as a health behavior. Risk communication is broadly thought of as conveying written, verbal, or visual content about risks to an intended audience [16,17]. The primary goals of risk communication can include conveying the meaning of a risk, the level of risk, or other information about a risk to motivate “decisions, actions, or policies aimed at managing or controlling” risks [16]. For the purposes of this review, risk communication comprised any content delivery for the purpose of motivating a change in household well water testing behavior. Ideal risk communication development should thoroughly investigate topics including, but not limited to, message content and clarity, message framing, mode of delivery, target audience composition, target audience knowledge and perceptions, and the relationship between the messenger and target audience [16,17]. Although various risk communication strategies have been shown to be effective for numerous health behavior problems (e.g., HPV vaccination, smoking behavior, HIV transmission) [18,19,20], data are sparse regarding the effectiveness of risk communication on well testing behavior specifically. Furthermore, risk communication development—when practiced by agencies without formal training in risk communication—can be an unsystematized and poorly defined process. Risk communications are frequently founded on technical expertise and expert perceptions of what the public should know, rather than on an understanding of the existing knowledge, perceptions, beliefs, and attitudes of the target population [19,21]. Poorly designed risk communications can lead not only to ineffective interventions, but can also lead to unintended consequences and new risks. Morgan, Fischhoff, Bostrom, and Atman go so far as to say that researchers and practitioners should “no more release an unproven communication on people than an unproven drug” [19]. Given the size of the at-risk population and the range and severity of health effects associated with poor quality water supplies, evidence-based risk communication is critical for developing effective interventions that support well owners in choosing pro-health behaviors such as regular well testing.

**Figure 1:**
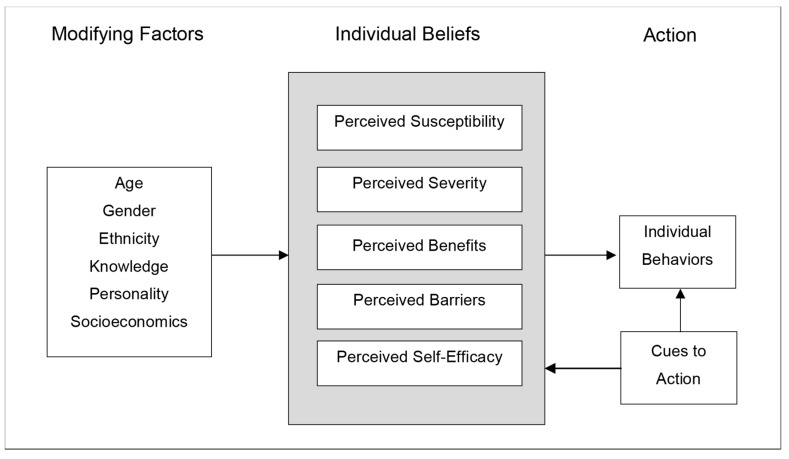
General components of the Health Belief Model (HBM). The HBM is a widely applied health behavior framework that can be used to explain and theorize areas to target in communication interventions. Source: Adapted from Glanz et al., 2015.

Understanding the cognitive and decision-making processes underlying a target behavior is critical for developing risk communications that promote the target behavior and ensure that the communications are appropriately targeted to a population [18,22]. Therefore, enumerating and conceptualizing the factors that may influence well testing behavior is a fundamental task for developing the evidence base needed to design effective risk communications to promote well testing behavior. The Health Belief Model (HBM) provides a useful organizing framework to conceptualize factors that may influence well testing behavior, and thus factors that might be considered when designing risk communications to effectively mediate behavior change. As Figure 1 shows, the HBM organizes factors influencing health behaviors into three major categories: (1) modifying factors, which are primarily demographic, structural, or socio-psychological variables that can influence behavior; (2) individual beliefs such as perceived susceptibility to a harm, perceived severity of a harm, perceived self-efficacy, and perceived benefits of or barriers to taking action; and (3) cues to action, which are events or phenomena that can trigger action [23]. The various factors within these categories can interact in many ways; for instance, increasing an individual’s perceived susceptibility may lower the threshold of an external cue needed to trigger a behavior change, or increasing the perceived benefits of a behavior may overcome the perceived barriers that inhibit action. The HBM is one of the most widely applied health behavior theories [18,24]. Although numerous, useful health behavior theories exist, the HBM was selected due to its emphasis on ‘cues to action’ [18]. Cues to action appear to be particularly important in stimulating testing behavior and thus provide a critical area for further examination [25]. Proponents of the HBM argue that “messages will achieve optimal behavior change if they successfully target” aspects of the HBM [24]. The HBM has been used to theorize and explain numerous health behaviors including vaccination behavior and health screening behaviors (e.g., breast cancer self-examination, Tay-Sachs genetic screening, participation in hypertension screening, etc.) [26]. The HBM has also been used to successfully design health behavior interventions; a meta-analytic review of tailored interventions to promote breast cancer screening found that interventions that used the HBM in their development were the most effective in promoting the target health behavior, suggesting that the HBM could be an effective framework for designing interventions in other health behavior scenarios [27]. Only one prior study has broadly used the HBM in the context of private well owners, using the model to gauge well owner risk perceptions [25]. Straub and Leahy suggest that the HBM could provide “a reliable theoretical approach to pro-environmental behavior modification” [25].

The overall objective of this research was to conduct a systematic scoping review to evaluate the existing evidence base for risk communications intended to promote the testing of private well water in developed nations. Specifically, we wanted to identify whether any studies had evaluated the effectiveness of risk communications in promoting private well testing in developed nations, that is, whether any studies had quantified the impact of risk communications on the rate of private well testing. We also sought to identify studies that assessed factors associated with private well testing behavior that may inform the development of effective risk communications. We synthesized the identified well testing factors into the HBM to facilitate future development of evidence-based risk communications for private well testing.

Although a prior descriptive overview by Morris et al. [28] addressed similar topics, this article is the first to use a Preferred Reporting Items for Systematic Reviews and Meta-Analyses (PRISMA) framework. The original PRISMA framework served as an important step in establishing a widely accepted, evidence-based standard of quality for systematic reviews [29]. The PRISMA Extension for Scoping Reviews (PRISMA-ScR) serves as a standard for knowledge synthesis in cases where full systematic reviews are not feasible or appropriate, as is the case for this scoping review of a topic without consistent summary measures [30]. To our knowledge, this review is also the first to enumerate relevant factors identified in the literature, such that researchers and practitioners can more readily identify areas with extensive evidence and areas that require more investigation. Furthermore, the HBM has not been previously used to inform the design of well owner behavioral interventions such as risk communications that are intended to help private well owners monitor and manage their water quality. The use of a well-established health behavior model may facilitate the translation of research into practice for a given population. Thus, this article presents a more systematized scoping review, an actionable way of synthesizing relevant data, and may enable implementation of evidence-based interventions.

## 2. Materials and Methods

### 2.1. Text Selection

To identify peer-reviewed studies assessing risk communication to promote private well testing and factors influencing well testing, a scoping review was performed following the PRISMA-ScR [29,30]. Three databases were searched: PubMed, Scopus, and Web of Science. Search terms (Table 1) were developed to capture results that pertained to the three main components of the research questions: communication, private wells, and testing.

The three databases were originally searched on 9 February 2018 and the results were refreshed on 27 March 2019. No other search restrictions such as publication date, funding sources, or institutional origin were in place. The three sets of search results were imported into Covidence, a web-based software capable of facilitating a systematic review framework. Covidence was used to remove duplicates from the resulting combined database.

Inclusion criteria (Table 2) were developed to identify studies in the search results that would be pertinent to the research objectives. Studies had to be in English, take place in a developed country according to United Nations classifications [31], mention a risk communication intervention to encourage voluntary private well testing or factors influencing decisions to test private wells, and have been published in a peer-reviewed journal. Studies involving well testing behavior in developing countries are abundant in the literature, but were excluded due to contextual differences (e.g., availability of testing infrastructure, literacy rates, income, etc.) that may preclude clear data synthesis.

Two reviewers independently screened the resulting texts against the inclusion criteria. Papers were initially screened by title and abstract for compliance with the inclusion criteria, and qualifying texts were moved to a secondary, full-text screening. The full texts of the qualifying studies were independently reviewed to further assess compliance with the inclusion criteria. Reference lists of any previous systematic reviews included in the full-text review were also screened in the same manner to identify any relevant studies not captured by the initial database search [28]. The resulting independent lists of qualifying texts were then reconciled between the two reviewers. Consensus was eventually reached for all documents. Potential risk of bias across studies was also considered (see section titled *Risk of Bias*).

### 2.2. Data Extraction and Synthesis

Data were extracted through a vote-counting procedure that has been used in previous public health-related systematic reviews [32,33,34]. This vote-counting procedure, adapted from Lewis and Pattanayak (2012), requires the identification and categorization of factors potentially influencing the outcome of interest (in this case, testing of private well water quality). This provides a useful basis to quantify the different factors associated with private well testing behavior present in the existing literature and estimate the directional influence of a factor on the target behavior. Many of the studies were qualitative or descriptive in nature; they reported, for example, the prevalence of beliefs, crude rates, or proportions of water stewardship practices, and/or interview responses. Therefore, factors could be categorized as: (1) positively or negatively associated with well testing behavior, and (2) statistically or not-statistically significant. A screener recorded votes for the direction and significance of a factor’s relationship with well testing behavior. Each paper could only contribute one vote to each factor category.

Factor categories were developed in the context of the HBM. Factors described in the literature were classified as either an individual belief (i.e., perceived susceptibility, perceived severity, perceived benefit, perceived barrier, or perceived self-efficacy), a cue to action, or a modifying factor. Factors and their vote-counts were grouped into these broader ‘bins.’ A factor codebook was developed based on an initial reading of the selected studies and factors were subsequently coded according to these categories.

## 3. Results

### 3.1. Overview of Studies

**Figure 2:**
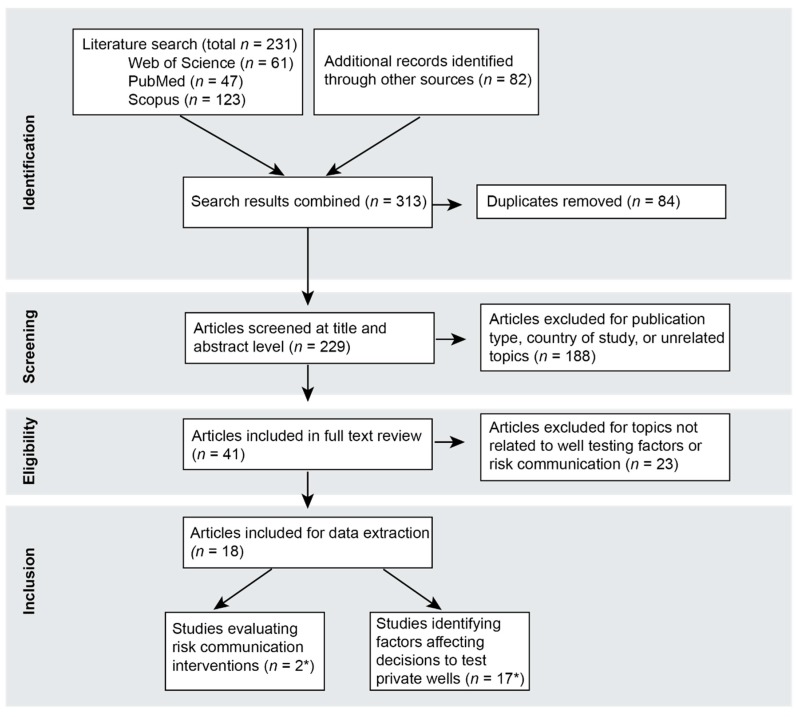
Preferred Reporting Items for Systematic Reviews and Meta-Analysis (PRISMA) flow diagram for the stages of selecting articles for inclusion. * One article addressed both risk communication interventions and factors affecting decisions to test private wells.

The systematic scoping review identified a total of 313 studies, of which 18 were ultimately selected for data extraction and synthesis (Figure 2). Sixteen of the studies addressed factors influencing well testing behavior, one study addressed the effectiveness of risk communication interventions, and one study addressed both. The included studies were published between 2005 and 2018 and ultimately included research conducted solely in North America, with most studies occurring in the northeastern United States or eastern Canada (Table 3).

Well testing rates varied across studies, but in general did not comply with the rates that local health departments recommend. Well owners who had tested at least once for any analyte ranged from 31% to 94%. Well owners who reported testing either annually or in accordance with their local, state, or provincial recommendations ranged from less than 6% to a high of 59%; however, this rate was typically less than one-third. The maximum annual testing rate (59%) represented a small focus group study (n = 22) drawn from a population that was “more likely to have well owners who performed annual testing than other townships” due to a prior well testing intervention in that township [35]. Table 4 presents a summary of the well testing rates for studies that included this information.

In general, study participants trended toward older, higher-income, and more highly educated populations when these data were reported. This is further discussed in the section on *Risk of Bias.* Select demographic information is presented in Supplementary Table S1.

### 3.2. Empirical Studies of Risk Communication Effectiveness

Two identified studies evaluated the effectiveness of risk communication interventions. Both studies focused on strategies to increase arsenic testing in areas with naturally elevated arsenic levels: Eastern Townships, Quebec, Canada and Tuftonboro, New Hampshire, USA.

The Quebec study, conducted by Renaud et al., employed two interventions to increase arsenic screening in well water: (1) a mass media campaign, which included a press release, television interviews, newspaper coverage, and online information related to arsenic, and (2) a community-based intervention in conjunction with the mass media campaign, which also sent arsenic information sheets to well owners through targeted mailings, added a well test reminder to tax documents, distributed arsenic information to new property owners, and partnered with local healthcare providers and environmental laboratories to increase the distribution of the arsenic information sheet. Phone surveys were conducted following both the mass media and community-based interventions to evaluate the respondents’ recall of their exposure to testing information and whether they subsequently performed an arsenic test. The mass media intervention increased the odds of arsenic testing by 1.73 (95% CI: 0.69−4.36) when compared to the unexposed control population; however, this increase was not statistically significant. Residents that were exposed to both the mass media and community-based interventions had statistically significantly increased odds of arsenic testing (OR: 4.79, 95% CI: 1.99−11.49, *p* < 0.001) compared with those who were unexposed to both. The authors did not calculate the well testing effect of exposure to the community-based intervention alone. Thus, Renaud et al. suggested that risk communication strategies can increase the rate of private well testing; however, the exact mechanisms for the effectiveness of this intervention are still largely undefined. Renaud et al. hypothesized that the delivery of risk communications directly to private well owners’ homes and their relationships with local environmental laboratories, healthcare professionals, and the municipality were likely to be responsible for this intervention’s success.

The New Hampshire study, conducted by Paul et al., introduced a collaborative well testing initiative that engaged numerous state and local agencies and academic institutions as partners [36]. The initiative primarily centered on a “well testing drive,” where volunteers transported well testing kits, well water samples, and test results to and from the well owners’ residences, thereby removing the inconvenience factor associated with well testing. The initiative also incorporated risk communications including local media coverage of high arsenic concentrations in local well water, posters about the well testing initiative in post offices and libraries, and notifications sent out with tax bills. Success of the intervention was measured by evaluating the number of well water samples submitted to the state’s environmental analysis laboratory pre- and post-intervention. Although this study design could not demonstrate causality, the post-intervention period presented a marked increase in the number of well water samples from the intervention area. Following the first round of the intervention in 2012, 159 samples were analyzed, and an additional 190 samples were analyzed after the second round in 2013. During the previous six years (i.e., pre-intervention period), a total of 83 samples had been processed, an annual average of 14 samples. This study, while demonstrating promise, has potentially conflated the influence of other factors in decisions to test wells such as the factors of cost and inconvenience (See the *Perceived Barriers* section).

The two studies’ interventions included numerous risk communication strategies and modes of delivery, but did not provide reproductions of the distributed materials, did not describe message content or framing, and did not describe how the communications were developed. Given the study designs and reporting methods chosen by the authors, it is not possible to draw clear conclusions about specific aspects of the risk communication interventions that facilitated their effectiveness and could be adapted for other populations. This presents a significant data gap for future researchers to address.

### 3.3. Factors Influencing Well Testing Behavior

Factors influencing well testing behavior identified in the studies were numerous and complex. As described in the *Methods* section, identified factors were coded and categorized in the context of the HBM. The final factor codebook is presented in Supplementary Table S2 and a summary of the factors extracted from the articles is presented in Supplementary Table S3.

#### 3.3.1. Modifying Factors Associated with Well Testing Behavior

**Figure 3:**
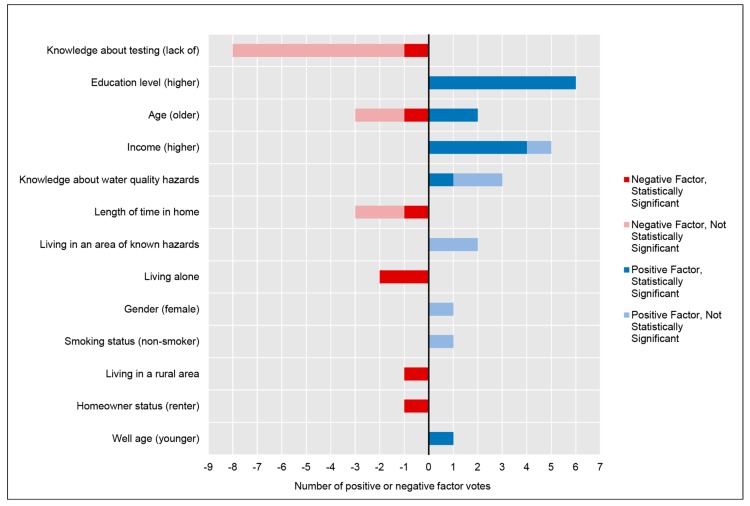
Counts and associations of modifying factors identified in the literature. Modifying factors are primarily demographic, structural, or socio-psychological variables that can influence behavior. Factors are presented in order from the most prevalent to least prevalent in the literature.

Thirteen modifying factors were found to be either positively or negatively associated with well testing behavior; these factors and their prevalence throughout the selected studies are presented in Figure 3.

A lack of knowledge about testing (e.g., how to test, where to test, what analytes to test, when to test, how often to test, etc.) was one of the most common factors associated with well testing. Lack of knowledge about testing was consistently negatively associated with testing behavior, although only one study presented a statistically significant negative association. In one study, only 8% of respondents correctly identified the local health authority’s recommendation for well testing frequency [12], while a separate study found 18% did not know how to test their water [11]. ‘Lack of information on testing’ was among the top five reasons for well owners not testing in one study [37], while responses such as ‘I don’t know how to have my well water tested’, ‘I don’t know what to test for’, and ‘I did not know testing was available’ were among the top reasons for not testing in a separate study [8]. These survey trends were reaffirmed by studies that interviewed participants: one study participant stated “*I don’t really know what all [testing] entails…. I don’t know how to get it tested*” [10] and a separate study’s participant stated “*I mean, I’ve lived out there for...years and I didn’t realize that there were places that you could take your water to get tested, or whatnot. So the awareness is definitely not there ...If I don’t want to get off my lazy [behind] and go and do [the testing], that’s my choice. [But] right now, I didn’t know I had a choice*” [38]. Lack of knowledge about testing may also serve as a barrier when the choices associated with testing are too abundant and the well owner lacks clear guidance, for example, in the selection of analytes given that relevant analytes may vary regionally. Many well owners suggested that outreach to them should include guidance on the analytes recommended for their area. One interviewee stated “*I would like to know... what else I could have my water tested for, and maybe should have my water tested for, depending on the area I live in.*” [38]. Previous psychosocial research supports the need for unambiguous guidance in this area; the presentation of too many choices can cause consumers to question the benefits or risks associated with each choice, induce feelings of being psychologically overwhelmed, and ultimately lead them to decide on no action [39]. Overchoice, a commonly documented phenomenon in behavioral economics, may be a factor to consider minimizing in private well owner outreach.

A higher education level and higher income were both prevalent modifying factors and commonly showed positive, statistically significant associations with well testing behavior. These important modifying factors may also mediate other factors in the HBM such as knowledge about water quality hazards, which was an infrequent yet positive factor. For example, one study found that knowledge of well water as a source of arsenic exposure and the knowledge that well water quality could change over time were significantly predicted by income and education [40].

The length of time a resident has lived in their home also exhibited another potentially important negative factor. One study showed that the longer a resident lived in their home, the less likely they were to have tested their well in the past five years [41]. Some studies have suggested that residents may succumb to a sense of complacency and lack of concern after many years in a home without any detected water quality problems (See section *Individual Beliefs Associated with Well Testing Behavior*), although there are likely to be other factors involved. Living alone was also strongly and negatively associated with conducting well tests [42,43], although the studies did not offer explanations about why this might be a factor. Potential causes could relate to a cost or inconvenience barrier, a lack of perceived susceptibility since only one person would be at risk, or an unidentified social norm.

Participant age showed contradictory results and was inconclusive as a modifying factor. This ambiguity may derive from differences in the underlying sample populations. Less prevalent modifying factors included gender [8], smoking status [8], well age [9], and rurality of the area [12].

#### 3.3.2. Individual Beliefs Associated with Well Testing Behavior

**Figure 4:**
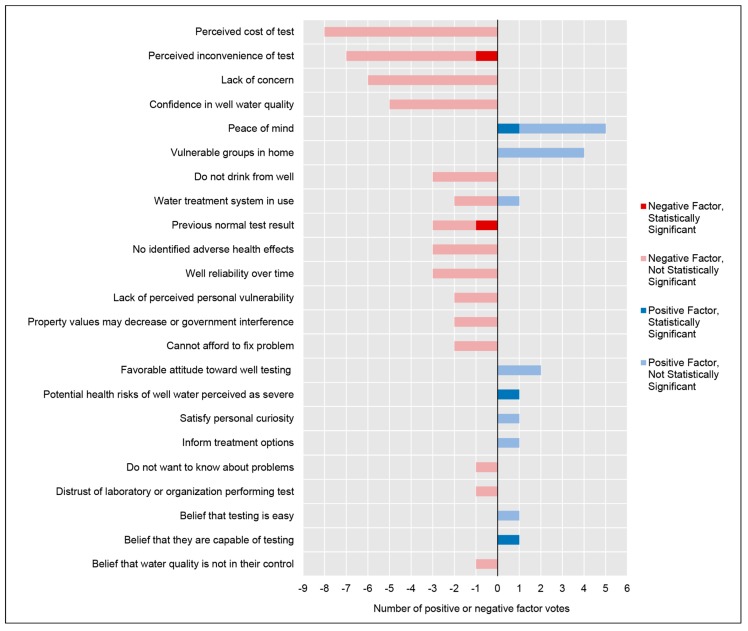
Counts and associations of individual beliefs identified in the literature. Individual beliefs that can influence behavior include factors related to perceived susceptibility to a harm, perceived seriousness of a harm, perceived self-efficacy, and perceived benefits and barriers to taking action. Factors are presented in order from the most prevalent to least prevalent in the literature.

Beliefs and perceptions were the most frequently cited factors associated with well testing behavior. Twenty-three beliefs or perceptions were found to be either positively or negatively associated with well testing behavior; these factors and their frequencies are presented in Figure 4. The broader category, Individual Beliefs, has been subdivided according to the HBM: Perceived Susceptibility, Perceived Severity, Perceived Benefits, Perceived Barriers, and Perceived Self-Efficacy.

##### (i) Perceived Susceptibility

Perceived susceptibility broadly relates to an individual’s assessment of the likelihood of an adverse event occurring [18]. Factors in this subcategory mediate whether a well owner believes their well could feasibly pose a risk to them. For instance, the perception that the likelihood of an adverse effect is high may move them to action (i.e., test their well), while the perception of the low likelihood of an adverse effect may inhibit action. Importantly, most factors in this subcategory were negatively associated with well testing, suggesting that many well owners do not perceive a high likelihood of adverse events due to their well, despite relying on potentially unfounded or misguided assumptions in their likelihood assessment. For instance, the factors of ‘confidence in well water quality’, ‘a previous normal test result’, ‘well reliability over time’, and ‘no identified adverse health effects’ were very common reasons for not testing a well; however, many participants were not correspondingly aware that water quality can change over time or that adverse effects may take years of cumulative exposure. These factors are likely to be related to the modifying factor of ‘knowledge of water quality hazards’, and also appear to be related to the important cue to action factor ‘a change in aesthetics’ (see section *Cues to Action Associated with Well Testing Behavior*), whereby well owners appear overly reliant on sensory perceptions to assess risks or the likelihood of risks from their well water. The perception of a status-quo condition—with respect to health, aesthetics, or test results—may prevent well owners from experiencing the perception of susceptibility needed to take action. For example, one study’s interviewee claimed, “*You get a couple of good readings over and over and over again that you’ve got fantastic water, and after a while I say ‘oh, well, it’s fantastic water’. Why am I going to waste my time with all this?*” [38], reaffirming the results of a separate study that found a lack of prior contamination significantly reduced the odds of well testing (OR: 0.37, 95% CI: 0.24–0.58) [9]. Another study identified that the top reason for a well owner’s confidence in their water quality was previously receiving an acceptable test result [37]. Furthermore, an interviewee stated “*We went probably 50 years and never tested it. And it always tastes good, so we assume that it’s alright*” [35] while another stated “*I just don’t feel there’s any [reason to test]—it’s a good well, there’s nowhere that there’s runoff, there’s no reason that I could think that it would be contaminated. None of us are sick. So…maybe someday [I’ll test]*” [35]. These statements also echo survey results, where one study found nearly 46% of survey respondents agreed that ‘our water is probably fine’ and 66% stated ‘they have been drinking well water for years without any problem’ [8]. The prevalent perceptions of status-quo conditions are concerning given the low testing rates observed across studies and tenuous levels of knowledge and health literacy regarding water quality hazards. The potentially unfounded confidence in well water quality was evident across these studies and is further discussed in the section *Cues to Action Associated with Well Testing Behavior*.

There was also an occasional lack of perceived personal vulnerability even when a well owner was broadly aware of water quality hazards, but demonstrated either a lack of deeper understanding of water quality that prevented them from feeling susceptible, or a general lack of perceived personal vulnerability that could be associated with an optimism bias. One study demonstrated that half of the interviewees readily identified a specific contaminant as a concern for the general area, but did not identify the contaminant as a risk at their hyper-local level, with one interviewee stating “*Well it’s not a problem on this road here but in some of the homes right on the water they have some problems*” [12]. Another study found that most respondents agreed that a local contaminant posed a severe health threat and that it could be found in water, but felt a low personal vulnerability to that threat [41].

The factor of ‘water treatment system in use’ was primarily negatively associated with well testing, although one study showed a positive association. The belief that a water treatment system is sufficiently protective against well water risks may or may not be unfounded, depending on the water quality and the treatment system in use. Across studies, when those who treated their well water were asked what treatments they employed, the overwhelming answer was ‘water softeners’ [8,11,37,38]. Water softeners improve the aesthetic qualities of the water, but typically do not protect against microbial or chemical hazards [8]. Thus, well owners may erroneously believe that well tests are not necessary due to ongoing treatment of some kind, whether or not the treatment is appropriate for their own water quality conditions. Similarly, studies have documented well owners who reported that they did not test their well because they did not drink from it [8,10,37]. Although this seemingly protective behavior may protect against some risks, it does not address the dermal and inhalation exposure pathways, does not entirely eliminate the ingestion pathway, and could therefore provide an erroneous sense of protective behavior.

##### (ii) Perceived Severity

Perceived severity relates to beliefs about the consequences of not practicing a particular health behavior (i.e., not testing well water) [18]. This subcategory was the least frequently encountered in the literature. Perceived severity is likely to be related to modifying factors such as ‘education’ and ‘knowledge about water quality hazards’. For instance, well owners with at least a bachelor’s degree had stronger perceptions of arsenic as a severe health threat, and correspondingly, those with higher levels of education were more likely to test their wells [40]. The perceived severity of health threats posed by well water necessarily requires basic knowledge about water quality hazards and health literacy as antecedents.

Vulnerable groups such as children, the elderly, and immunocompromised may experience particularly severe health outcomes or face higher risks compared to the general population [6,44]. Although the presence of vulnerable groups may additionally constitute a modifying factor, this factor is also intrinsically linked to the perceived severity of consequences associated with not practicing well testing. The identification of vulnerable groups in the home was indeed a positive factor for well testing [8,12,42,43]; however, vulnerable groups identified in the literature only specifically included children, thereby excluding the elderly, immunocompromised, and other potentially vulnerable groups. It is unclear whether well owners do not know that these other groups are considered vulnerable and did not independently identify them, whether well owners perceive children to be the most vulnerable group, or whether other groups were merely omitted by the researchers. Of note, well owners across these studies were generally older than the broader study area populations; it would be important for future researchers and practitioners to determine if elderly well owners perceive themselves as belonging to a vulnerable population.

A general lack of concern and a sense of apathy were frequently cited as reasons for not performing well tests. A lack of concern may be related to modifying factors such as ‘knowledge about water quality hazards’, or a well owner’s existing knowledge may not have imparted a sense of urgency. For instance, 60% of survey respondents in one study reported having ‘no concern’ or ‘low concern’ about the health risks of arsenic, despite living in an area with elevated levels of arsenic in groundwater [12]. Conversely, a separate study of an area with elevated NO_3_ levels found that well owners who responded that they were ‘very’ or ‘somewhat’ concerned about contamination were more likely to test their water, compared to those who responded that they were not concerned [11]. A general sense of apathy was also documented in qualitative studies, with one interviewee responding “*I’m lazy. I’m not worrying about it enough to do it*” [38], while a separate study’s interviewee responded they “just hadn’t gotten around to it yet”, despite that individual demonstrating knowledge of wells and contamination sources [10].

##### (iii) Perceived Benefits

Perceived benefits—that is, internal beliefs about the advantages of performing a particular behavior—may help overcome other factors that inhibit well testing behavior [18]. As would be expected, all identified perceived benefit factors were positively associated with well testing. The factor ‘peace of mind’ was the primary benefit cited within the literature and included studies with statistical significance. ‘Peace of mind’ was the top reported reason for testing a well in numerous studies [8,9,35,41], with one study reporting statistically significant odds of annual testing (OR: 4.84, 95% CI: 2.54–9.25) among well owners who tested for that reason [9].

Less frequently documented benefits included satisfying a personal curiosity [9], informing water treatment options [8], and seeing well testing as a generalized benefit (i.e., a favorable attitude toward well testing) [35,40].

##### (IV) Perceived Barriers

Perceived barriers include any obstacles that may prevent someone from performing a well test including straightforward barriers such as cost and time, and less obvious barriers such as the perception of negative consequences as the result of performing the test [18]. Perceived and actual barriers to well testing have frequently been cited in the literature. The cost of well testing and the inconvenience of well testing were substantial barriers to routine well testing. The inconvenience of well testing included concepts such as ‘it takes too long’, ‘there are too few testing locations’, and ‘the testing location’s hours of operation are not convenient to me’. Convenience may be a particular challenge for rural communities, caretakers (e.g., parents or caretakers of the elderly), and those with fulltime jobs. The influence of this obstacle was documented both quantitatively and qualitatively. Survey respondents who disagreed or strongly disagreed with the statement “the test is convenient” had significantly reduced odds of testing their well annually (OR: 0.42, 95% CI: 0.26–0.66) [9]. Interviews revealed the personal cost of this inconvenience, with one fulltime farmer stating “*There’s only so many things you can do, so it usually doesn’t get done. That’s about the only way you can put it*” [35] and a parent stating “*I think what’s very awkward is, you know where I live in particular, what’s being asked was a very difficult thing to do because we are a working family with children, and to try to get water tested from a remote community more than an hour away from downtown, it was awkward to do that*” [12]. The barriers of inconvenience and cost appeared to work synergistically to inhibit action and were commonly mentioned together; one study removed both of these barriers by providing a free well test and sample pickup service and doubled the background testing rate [45]. Cost of well testing was also frequently cited as an independent barrier, despite these study populations frequently trending toward higher-income brackets. One study found that a $40 well test could reduce the demand for testing by 70%, as compared to a free test [43].

Less commonly cited barriers to testing included a distrust of the organization performing the test [37], a desire to not know about any potential problems [8], and the concern that a positive result may decrease property values or trigger government action against the well owner [25,38].

##### (V) Perceived Self-Efficacy

Self-efficacy relates to the belief in one’s own ability to perform a particular behavior or cope with the consequences of that behavior [18]. Mirroring the perceived barriers, the most frequent factor in the perceived self-efficacy subcategory related to cost and finances, in this case, the perception that a well owner could not afford to “fix” a problem if the test result indicated one was present. The perception that treatment or remediation was unaffordable was negatively associated with well testing behavior [8,38]. The perception that water quality is not in the control of the well owner—that is, that water quality is dependent on regional characteristics or activities and cannot be changed—was also negatively associated with well testing [8]. The beliefs that well testing is easy and that a well owner is capable of testing their well were both positively associated with well testing behavior [40,43].

#### 3.3.3. Cues to Action Associated with Well Testing Behavior

**Figure 5:**
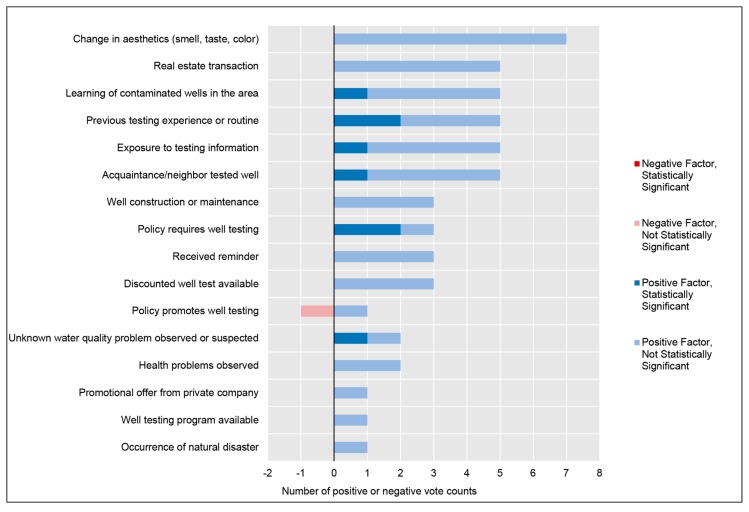
Counts and associations of cues to action identified in the literature. Cues to action are internal or external events or phenomena that can trigger action. Factors are presented in order from the most prevalent to least prevalent in the literature.

Cues to action involve internal or external events that instigate a health behavior [18]. It has been debated how cues to action interact with other aspects of the HBM, with some claims that perceived benefits or perceived susceptibility only become psychologically relevant enough to elicit action in the context of a triggering event [18]. As expected, nearly all identified cue to action factors in this scoping review were positively associated with well testing behavior. Factors related to cues to action are presented in Figure 5.

Foremost among the cue to action factors was a change in aesthetics such as a change in the odor, appearance (e.g., color, cloudiness) or taste of the well water. When asked what would cause them to test in the future, one study’s participants overwhelmingly selected ‘a change in taste, smell, or appearance of my water’, (76% of respondents), far surpassing other seemingly important cues to action such as ‘unexplained health problems’ (36%) or ‘learning that some wells in my town are contaminated’ (31%) [41]. Similarly, 80% of a separate study’s participants selected a ‘change in taste, smell or appearance’ as a reason that would prompt them to test in the future [42]. A qualitative study reported that all interviewees mentioned some level of reliance on aesthetics in detecting contamination [10], while another study’s interviewee stated “*Oh, I’m not all that diligent in checking my water. As long as it tastes good, that’s fine*” [38]. Across and within studies, sensory perceptions appeared to play a critical role in mediating well testing behavior and intentions to test in the future. Given that most contamination cannot be detected through the senses, it appears that there may be a large population of ‘dormant’ well testers waiting for a testing trigger that is unlikely to come.

Cumulatively, well testing policies—and relatedly, real-estate transactions—seem to be important cues to action; however, there appears to be a distinct difference between policies that require well testing and policies that merely promote well testing. One study examined the effect of a state-level policy in New Jersey that required a well test at the point of sale for residential homes and determined the policy increased the arsenic testing rate from 35% of households to nearly 100% of households; however, it should be noted there is a known recall bias of underreporting, in that only 60% of tested households actually recalled that they had tested their water, despite evidence of their arsenic test in state databases [42]. The real-estate requirement also appears to have tangential well-stewardship benefits such as significantly higher rates of treatment, higher perceptions of vulnerability to risks associated with water contamination, feelings of a greater personal obligation toward testing, and intention to test within the next year [42]. A separate study in Ontario, Canada found that a legal requirement increased the odds of annual testing by a factor of 2.43 (95% CI: 1.19–4.98) [9]. A real-estate policy in Oregon that did not have enforcement or outreach mechanisms failed to achieve the significant benefits observed with the New Jersey real-estate policy; in fact, well water sample submissions associated with the Oregon policy decreased over time despite increasing real-estate transactions, and the Oregon policy is now viewed as ‘voluntary compliance’ [46]. Regardless, general real-estate transactions without associated policies still appear to be important triggers for well testing events. One study identified that mortgage lenders requiring a well test was an important prompt [12], while other studies identified simply that the act of buying or selling a home was a common reason for testing a well [8,35,41,42]. Despite these positive associations, it appeared such well testers were less likely to recall ever testing their well, what they tested for, or what their results were [42]. These processes may be handled by third parties and/or otherwise disengage well owners from the well testing process and from their water quality more broadly.

Social networks such as neighbors, friends, and acquaintances appear to be important sources of information, provide cues to action for well owners, and may be a source of social norms surrounding well testing behavior. One study demonstrated that arsenic screening was 11 times more likely among well owners who reported that they knew an acquaintance who had already screened their well for arsenic [47]. Seventy-four percent of another study’s participants said learning that their neighbors had contaminated water would prompt them to test their own well, a proportion not much lower than observing a change in their own water quality (80%) [42]. Another study’s interviewee stated “*...there was a lot of talk about some of the houses right down in the village having problems, so it was close enough, hearing about local problems, we figured we’ll test ours and see what it is*” [38]. As alluded to in the preceding quote, the proximity of well water concerns appears to have a gradient effect, with fewer people reporting that contaminated wells in their town or in the general vicinity would prompt them to test their own well. Only 40% of respondents agreed that ‘learning that some wells in town have contaminated water’ would prompt them to test their own well, compared to the 74% that would test if a neighbor had contaminated water and 80% that would test if they observed a change in their own water [42].

Although prior testing experience may inhibit action in some cases (see *Perceived Susceptibility* section), prior testing experience may act as a cue to action when well owners view the act as a routine or chore. Kreutzwiser et al. found that the odds of annual testing were substantially increased when the reason for testing related to a routine (OR: 11.70, 95% CI: 6.04–22.68) [9]. While previous normal test results may inhibit future intentions to test, previous abnormal test results may serve as a trigger for developing a testing routine, with one interviewee stating “*... when we failed the test, that’s when we upped the ante a bit in terms of getting it tested*” and other interviewees mentioning a previous abnormal test result as the reason they tested regularly [38].

The effectiveness of exposure to testing information and/or receiving reminders is unclear, largely due to the scarcity of experimental studies testing these interventions (see *Empirical Studies of Risk Communication Effectiveness* section), although the current literature appears positive overall. One study observed that well owners who lived in high-intervention areas (i.e., repeated well owner outreach) were more likely to test their well (OR: 1.63, 95% CI: 1.01–2.63), although this was not a causal relationship between exposure to information/reminders and testing behavior [43]. Many participants viewed reminders positively, with some well owners suggesting that a reminder would help them test in the future [25] or that reminders like flyers and brochures would be good methods to increase well testing rates in their community [37]. Several focus group participants agreed that reminders could help annual testing become more routine, comparing it to the common cultural association of annual smoke alarm checks and Daylight Savings Time: “*It’s like, you always hear about it and you think, okay, change your clock, you think about your [smoke alarm] battery. It becomes, it’s like advertising or something... it becomes part of your consciousness over time*” [38].

### 3.4. Risk of Bias

No individual articles were excluded due to risk of bias, although there is risk of bias across all studies. First, there is evidence of publication bias; due to the small pool of researchers working on these specific topics, there is some overlap in authorship among the studies—four of the studies had the same first author, and other articles shared co-authors. There is also the potential for selection bias among study participants. Study participants typically had a median age above 50. Furthermore, household incomes frequently appeared higher than the general population from which they were selected—several studies’ median household incomes were around or above $USD 100,000 per year [12,37,42,43]. Only one study specifically investigated low income residents [6], who may comprise a key demographic of well owners depending on the setting. Although race/ethnicity was an infrequently reported demographic, most study participants were drawn from predominantly white populations, and only one study demonstrated a particular focus on non-white populations [10]. The well owners captured in these studies additionally appeared to be highly educated, with most having at least some college education when this variable was reported by researchers [12,41,42,43]. These potential selection biases may fail to capture the experiences, perceptions, motivations, and barriers of other well owners, which may under or overestimate the true frequency of factors in the general population and in marginalized communities who may experience environmental justice issues. Key demographics for the selected studies are summarized in Supplementary Table S1. Despite the potential risk of biases, these studies present significant and substantial contributions to the literature, help identify data gaps where additional research or effort is needed, and provide an initial framework for researchers and practitioners to adapt to their own settings.

## 4. Discussion

### 4.1. Summary of Evidence

**Figure 6:**
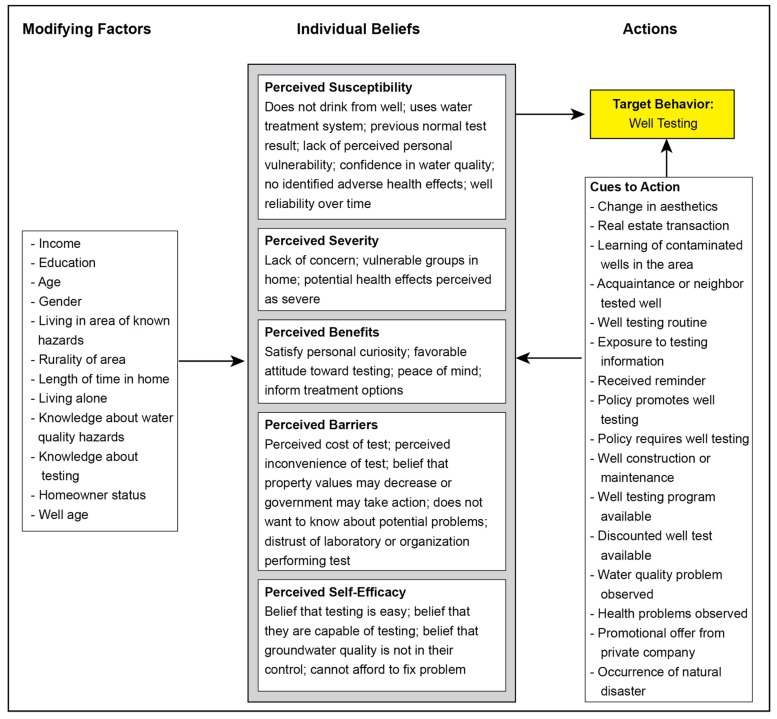
Updated Health Belief Model (HBM) with synthesized data from the systematic scoping review. Source: Adapted from Glanz et al., 2015.

This systematic scoping review descriptively synthesized data from a selection of quantitative and qualitative studies to (1) evaluate the effectiveness of existing risk communications intended to encourage testing of private well water, and (2) better define the factors feasibly involved in the decisions by well owners to engage in water testing. Figure 6 presents an updated schematic of the HBM with factors identified from this scoping review. This mixed-methods approach provides dimension and context that can be difficult to capture with quantitative studies alone, while the quantitative studies provide analytical rigor. The appearance of a factor in both qualitative and quantitative studies lends weight to the idea that it may be important.

The two empirical studies identified in this review exhibited positive results; however, the scarcity of studies examining the effectiveness of risk communications among private well owners prevents well-defined conclusions or trends. Risk communication considerations are multifaceted (e.g., mode of delivery, message content, audience framing, etc.), and the shortage of studies and differences in reporting practices among existing studies precluded more detailed analyses on specific aspects that facilitated effectiveness. Although the quasi-experimental studies of risk communications described were insightful, future studies should consider randomized control trials to test the effectiveness and determine causal relationships in the exposure of well owners to risk communications and their testing behavior. Furthermore, neither Renaud et al. nor Paul et. al. provided detailed descriptions or copies of the materials used in their respective risk communications within their articles or supplementary materials; descriptions were limited to broad terms such as “articles”, “posters”, “flyers”, and “leaflets”. Informational content, graphic design, source credibility, and audience appeal are likely to be pertinent aspects of effectiveness and should be well-defined in the future, so that the emerging evidence base for risk communications to encourage well testing behavior is more transparent and easily adapted to other settings.

The HBM posits that individuals are likely to engage in a pro-health behavior if they believe they are susceptible to a threat if they do not take action, if the threat is sufficiently serious, if the pro-health action has clear benefits, if the barriers to taking action or costs associated with taking action are not too high, and if some event triggers them to perform the action [18]. These factors are all critical to consider in designing future risk communications; however, it is likely insufficient to target single identified factors or even single categories within the HBM [26,48]. Instead, aspects of the HBM interact with and mediate each other. Future practitioners and researchers should rigorously study and understand their intended beneficiaries, use the HBM to theorize factors that might interplay to produce behavior change in their population, and develop population-specific causal pathways in the context of the HBM. The factors presented here may help inform survey instruments to better understand the communities’ demographics, beliefs, and motivations prior to designing a risk communication. Conversely, if the community and intended beneficiaries are already well known and are engaged as stakeholders in the risk communication design, the factors presented here can be used as a framework to help tailor the communication to maximize its effectiveness in that community.

Modifying factors such as income and education were some of the most numerous and statistically significant predictors of well testing behavior identified in the included articles. Modifying factors, although typically not mutable, can provide indications of where public health professionals and risk communicators should focus their efforts to reduce disparities. For instance, Flanagan demonstrated that those who chose to participate in free and reduced-cost well testing initiatives were of higher income and higher education than the broader study population [43]. Such results, and the frequency of factors related to income and education, suggest that wealthier well owners tend to disproportionately benefit from outreach and interventions; traditional interventions including risk communications may unintentionally exacerbate disparities associated with well water and water quality hazards. Thus, very intentional outreach into low-income and lower-education communities is warranted, and risk communications should be designed appropriately.

Future risk communications should carefully seek to disabuse well owners of some of the misconceptions identified herein and/or accentuate aspects that encourage testing, as appropriate for specific populations. For instance, many well owners were confident in their water quality, despite infrequent testing and invalidly relying on their sensory perceptions to detect contamination. A recent review of factors influencing perceptions of private well water quality in North America identified well water quality perceptions as an important consideration in well water testing behavior, upholding this aspect of our findings [49]. Individuals appear to be more motivated to test their own well if they learn that a neighbor has tested than if they learn that wells in their town/vicinity are actually contaminated. Due to subsurface variation, this is a tenuous proxy, and residents may be underestimating their own vulnerability because of it. Similarly, previously normal test results appear to inhibit future intentions to test wells. Future risk communications may consider emphasizing the fact that water quality can change over space and time and contamination cannot typically be detected through the senses. Conversely, well owners appear to be positively affected when they view testing as a routine or if they are seeking peace of mind. Future risk communications that encourage aspects of a routine (e.g., yearly dentist appointments, yearly car inspections, yearly smoke alarm tests) and/or encourage well owners to seek peace of mind may be effective. Risk communications should also clearly specify the basics about well testing to increase the essential antecedent factor of “knowledge about testing” (e.g., how to test, where to test, what to test for in their given location, how often to test, etc.).

While they appear to be useful in triggering one-off tests (e.g., real-estate transactions), cues to action may not necessarily be conducive to long-term behavioral change. Cues to action could instead serve as effective time points for disseminating risk communications designed around factors that appear amenable to sustained behavioral change. For instance, targeted risk communication might be disseminated during the purchase of a home

Cost and inconvenience were two substantial barriers to testing. If local health departments or other community stakeholders cannot afford to remove these barriers entirely, they should consider ways to better facilitate testing in the context of those barriers and, at the very least, ensure future risk communications emphasize low-cost and ease of testing to reduce the perception of those barriers.

### 4.2. Limitations

This systematic scoping review relied exclusively on peer-reviewed literature in order to assess the current status of the academic evidence and to facilitate the development of evidence-based interventions. While this approach has its strengths and provided appropriate boundaries for the review, there is gray literature related to this topic that may also be useful in designing effective risk communications (e.g., county health reports and initiatives, conference proceedings, etc.). Future research could be undertaken to synthesize gray literature and compare the findings to this review. As with all systematic reviews, the search terms and databases may have excluded articles that would have indeed met the inclusion criteria. Furthermore, the scope of this review was restricted specifically to well testing behavior, although there is literature outside of this topic that would likely be insightful (e.g., cognitive psychology, behavioral economics, etc.). The scope and topic of this review necessitated a mixed-methods approach; none of the selected studies identified causal relationships between risk communication and well testing behavior. Significantly more research is needed in that area to perform a true systematic review or meta-analysis, whereby summary measures and summary conclusions can be made. Additionally, the risk of bias assessment identified that the published and selected studies may not be inclusive of all well owners, which introduces a limitation to this review. More research is needed to expand the demographic makeup of study participants to avoid exacerbating disparities and to capture the perceptions and experiences of all well owner populations. Furthermore, there was a lack of diversity in the types of contaminants in the included studies. For example, many studies involved testing for arsenic specifically, rather than well testing behavior more broadly. Risk communication techniques and factors that influence well testing behavior may differ depending on the relevant contaminants and associated risks for a given area, for example, arsenic risks may induce different behavioral responses than other chemical/metal risks, while chemicals/metals might induce different behavioral responses than microbial risks. Future research could elucidate any differences in approach that may be required for effective risk communications based on a population’s potential hazards.

The factor vote count is a useful technique for synthesizing mixed-methods studies and studies for which there is not a consistent outcome measure; however, this technique is not without limitations. Enumerating factors do not capture the ‘weight’ of the factors, that is, a factor’s significance or influence on well testing behavior. Factor vote counts simply capture the frequency of factors in the literature. The factor coding and extraction phase can also be subjective. The use of two reviewers helps to overcome subjectivity, but does not eliminate it entirely. Despite these limitations, this technique provides a useful foundation for future studies, in that it can identify where data gaps currently exist in the literature (e.g., poorly documented factors or factors that could potentially demonstrate causal or otherwise significant relationships) and thus where future research could be focused. For this reason, factor vote counting appears to be an appropriate scoping technique given the emergent nature of this specific field.

Well testing is merely the first target behavior in ensuring safe and reliable private water supplies. Although the scope of this review does not include subsequent steps such as methods for effectively communicating test results, well owner treatment behaviors, or well owner maintenance behaviors, it may still provide considerations for other related well stewardship actions. Notably, findings from several of the included studies suggest that treatment behaviors may be mediated similarly to testing behaviors. Jones et al. found many well owners “used treatment devices to decrease the hardness or sulfur content of the water, and not out of concerns for safety”, that is, treatment behaviors may also be connected to sensory or aesthetic qualities [38]. Malecki et al. identified reasons for well owners failing to treat or filter their water, which included factors similar to testing behaviors including aesthetics (‘our water does not smell or taste bad’, ‘our water looks clean’), perceived susceptibility (‘we’ve been drinking this water for years without any problems’), perceived barriers (‘it costs too much to filter or treat’), and knowledge (‘we do not have enough information about the subject) [8]. Testing and treatment behaviors may be linked to some extent (see *Perceived Susceptibility* section) and the actions may share some behavioral mechanisms. Therefore, additional study of the factors influencing treatment behaviors is warranted to further conceptualize the spectrum of well stewardship decision-making.

## 5. Conclusions

This systematic scoping review assessed the current evidence base relating to risk communications designed to promote well testing behavior. The review also identified factors that are associated with well testing behavior. Three databases were searched and 18 articles were ultimately identified as suitable for inclusion in the systematic scoping review; only two of which assessed the effectiveness of risk communication interventions. Although both studies found an increased prevalence of private well testing behavior in their study areas post-intervention, significantly more research is warranted in this area to develop the evidence base needed for rigorous, evidence-based risk communication design. While only two studies conducted empirical tests of risk communications, many more studies have elucidated factors influencing the decisions of well owners to test their wells. These factors were categorized and synthesized in the context of the Health Belief Model (HBM) and demonstrate areas where researchers and practitioners could consider focusing their future work. Risk communications designed in the context of a given population’s relevant HBM factors may yield tailored interventions that are community-appropriate, culturally accepted, and sustainable, thereby increasing the likelihood of establishing a population of routine well-testers and ensuring pertinent information is disseminated. The results from this systematic scoping review suggest that risk communications can be effective in motivating private well owners to test their water, that numerous factors interplay to inform well owner decision-making, and further investigations of the content, design, and delivery of risk communications is warranted. Such research is critical in ensuring that external stakeholders receive the optimal return on their outreach investments and that private well owners are effectively supported in their efforts to secure safe and reliable water supplies.

## Figures and Tables

**Table 1:** Search terms used in the study identification phase.

	“risk communication” OR “risk information” OR communication * OR promotion * OR education * OR outreach * OR “public relations” OR awareness OR campaign
AND	“private well” OR “private wells” OR “well owners” OR “private well owners” OR “water wells”
AND	test OR tests OR testing

* Asterisk indicates truncation syntax to allow the database to search for all forms of a root word

**Table 2:** Inclusion criteria used in the screening and eligibility phases.

	Inclusion Criteria	Exclusion Criteria
1. Language	English	Other
2. Study Location	Developed country	Developing country
3. (a) Intervention	Risk communication intended to encourage well testing behavior	Other
OR	OR	
(b) Factor	Factor that may influence well testing behavior	
4. Publication Type	Peer-reviewed journal	Other

**Table 3:** Characteristics of the included studies.

Author(s), Publication Year	Study Location(s)	Study Design	Total Sample Size *	Article Categorization	
				Empirical Risk Communication Assessment	Factors Influencing Decisions to Test
Chappells et al., 2015	Nova Scotia, Canada	Observational (mail survey, interview analyses)	420		**X**
Fizer et al., 2018	North Carolina, USA	Observational (interview analyses)	18		**X**
Flanagan et al., 2015	Maine, USA	Observational (mail survey)	525		**X**
Flanagan et al., 2016a	New Jersey, USA	Observational (mail survey)	711		**X**
Flanagan et al., 2016b	New Jersey, U.S.	Observational (mail survey)	670		**X**
Flanagan et al., 2016c	Maine, U.S.; New Jersey, USA.	Observational (mail survey)	1287		**X**
Hexemer et al., 2008	Ontario, Canada	Observational and Quasi-experimental (surveys and free well water tests)	97		**X**
Hoppe et al., 2011	Oregon, USA	Secondary Data Analysis	N/A **		**X**
Imgrund et al., 2011	Ontario, Canada	Observational (interview analyses)	22		**X**
Jones et al., 2005	Ontario, Canada	Observational (focus group analyses)	15		**X**
Jones et al., 2006	Ontario, Canada	Observational (mail survey)	246		**X**
Kreutzwiser et al., 2011	Ontario, Canada	Observational (mail survey)	1567		**X**
Lewandowski et al., 2008	Minnesota, USA	Observational (telephone survey and water quality testing)	483		**X**
Malecki et al., 2017	Wisconsin, USA	Observational (mail survey)	460		**X**
Paul et al., 2015	New Hampshire, USA	Quasi-experimental	N/A	**X**	
Postma et al., 2011	Montana, U.S.; Washington, USA	Observational (mail survey and water quality testing)	188		**X**
Renaud et al., 2011	Quebec, Canada	Quasi-experimental	539	**X**	**X**
Straub et al., 2014	Connecticut; Rhode Island; Maine; New Hampshire; Vermont, USA	Observational (mail survey)	714		**X**

* Response rate prior to any exclusions or other data filtering. ** 18,688 addresses.

**Table 4:** Summary of select well testing information from included studies.

Author, Year	*N*	Tested at Least Once for Any Analyte	Tests in Accordance with Local, State, or Provincial Recommendations or More Frequently	Tested for at Least One Analyte in Past 12 Months
Chappells, 2015	420	90%	12% ^a^	4% ^a^
Fizer, 2018	18	83%	<6%	<6%
Flanagan, 2015	419	78%	-	10%
Flanagan, 2016a	532	82% ^b^	-	15% ^b^
Flanagan, 2016b	670	82–83% ^c^	-	-
Flanagan, 2016c	972	~60–85% ^d^	-	-
Hexemer, 2008	97	-	-	25% ^e^
Hoppe, 2011	N/A	-	-	-
Imgrund, 2011	22	-	-	59%
Jones, 2005	15	-	Few ^f^	Few ^f^
Jones, 2006	239	79%	31%	23%
Kreutzwiser, 2011	1386	94%	-	35% ^a^
Lewandowski, 2008	483	-	29%	10%
Malecki, 2017	460	48%	-	19% ^a^
Paul, 2015	N/A	-	-	-
Postma, 2011	186	31%	-	-
Renaud, 2011	539	-	-	-
Straub, 2014	714	-	-	-

^a^ Among those who tested (when reported). ^b^ Data selected from the representative sample. ^c^ Range includes towns in all intervention groups. ^d^ Range includes low, medium, and high-income groups in Maine and New Jersey; total N is the summed number of well owners in all groups; data are presented on a bar chart, values are approximate. ^e^ Background testing rate for study population; likely includes well owners of various testing frequencies. ^f^ Data not provided: “…many participants reported that they had never tested their water, or only tested it once every few years”.

## Supplementary Materials

The following are available online at https://www.mdpi.com/1660-4601/16/22/4333/s1, Table S1: Summary of select demographic information from included studies; Table S2: Factor codebook; and Table S3: Summary of data extracted from included studies.
