# Supplementary material for: Risk Communication and Factors Influencing Private Well Testing Behavior: A Systematic Scoping Review
Source: Int J Environ Res Public Health. 2019 Nov 7;16(22):4333. doi: 10.3390/ijerph16224333 (PMC6888409; doi:10.3390/ijerph16224333)
Supplement: Supplementary file 1 [file ijerph-16-04333-s001.zip › Table S1_Demographic information from studies.pdf]

Table S1: Summary of select demographic information from included studies

Author, Year	N=	Annual Household Income ($USD*)		Education Level		Age (years)		Race/Ethnicity (% White)	
		Study participants	General study population	Study participants	General study population	Study participants	General study population	Study participants	General study population
Chappells, 2015[a]	420	43% ≥ $Can 100,000	NR	80% college/trade school or university	NR	54% ≥ 55 years	NR	NR	NR
Fizer, 2018	18	$62,500[b]	$63,791[c]	70% with associates degree or higher[c]	57% with associates degree or higher	11% ≥ 65 years[c]	9% ≥ 65 years	28%[c]	62%
Flanagan, 2015	419	$40,000-$59,000[b]	NR	Technical or community college[b]	NR	55 years[c]	NR	NR	NR
Flanagan, 2016a	532	$125,000-$150,000[b,d]	$121,095[b]	71% with Bachelor's degree	56% with Bachelor's degree	58 years[b]	51 years[b]	NR	NR
Flanagan, 2016b	670	$100,001-$150,000[b,e]	NR	60-75% with Bachelor's degree or higher[e]	NR	57-59 years[b,e]	NR	NR	NR
Flanagan, 2016c	972	NR	NR	NR	NR	NR	NR	NR	NR
Hexemer, 2008	97	NR	NR	NR	NR	NR	NR	NR	NR
Hoppe, 2011	N/A	NR	NR	NR	NR	NR	NR	NR	NR
Imgrund, 2011	22	NR	NR	NR	NR	NR	NR	NR	NR
Jones, 2005	15	NR	NR	NR	NR	NR	NR	100%	NR
Jones, 2006	239*	47% > $Can 70,000	31% > $Can 70,000	22% with university or graduate degree	16% with university or graduate degree	60% ≥ 50 years	41% ≥ 50 years	NR	NR
Kreutzwiser, 2011	1,386	NR	NR	100% with college/trade school or university	NR	67% ≥ 50 years	NR	NR	NR
Lewandowski, 2008	483	NR	NR	NR	NR	NR	NR	NR	NR
Malecki, 2017	460	22% > $99,999	19% > $100,000	37% with Bachelor's degree or higher	31% with Bachelor's degree or higher	34% > 60 years	23% > 60 years	NR	NR
Paul, 2015	N/A	NR	NR	NR	NR	NR	NR	NR	NR
Postma, 2011	188	82% < $49,999	NR	73% with college/trade school or higher	NR	33 years[f]	NR	92%	NR
Renaud, 2011	515	59% to 75% < $Can 59,999	NR	40% to 56% with college/trade school or higher[g]	NR	41% to 55% > 55 years[g]	NR	NR	NR
Straub, 2014	714	NR	NR	NR	NR	NR	NR	NR	NR

*$USD unless otherwise noted

NR - Not reported or not able to report

[a] Used overall study population characteristics

[b] Median value or category

[c] Subcategory is less than maximum N (when this information is reported)

[d] Data selected from representative sample

[e] Range includes median values from all intervention areas

[f] Mean value

[g] Range includes pre- and post-intervention respondents and intervention and non-intervention groups
