# Supplementary material for: Risk Communication and Factors Influencing Private Well Testing Behavior: A Systematic Scoping Review
Source: Int J Environ Res Public Health. 2019 Nov 7;16(22):4333. doi: 10.3390/ijerph16224333 (PMC6888409; doi:10.3390/ijerph16224333)
Supplement: Supplementary file 1 [file ijerph-16-04333-s001.zip › Table S2_Factor codebook.pdf]

Table S2: Factor codebook

Health Belief Model Construct	Factor Description	Factor Example
Modifying Factors	Person age (Older)	"Age is significantly and negatively associated with intention to test well water" - Flanagan, 2015
	Education level (Higher)	"Education is significantly and positively associated with having tested in the last 5 years" - Flanagan, 2015
	Gender (Female)	"Crude rates of testing in the last ten years verses not testing trended higher for females" - Malecki, 2017
	Income (Higher)	"Income is associated with greater appreciation for testing as a means to protect the health of one's family and stronger feelings of safety from having water tested... "Higher-income well owners are more likely to know who to contact for well testing and be confident in their ability to manage regularly testing and monitoring of water quality." -Flanagan, 2016c
	Smoking status (Non-Smoker)	"Crude rates of testing in the last ten years verses not testing trended higher for...non-smokers" - Malecki, 2017
	Living in an area of known hazards	"Respondents in higher risk areas were also more likely to have tested their well water at least once and to have tested for arsenic at least once." - Chappells, 2015
	Rurality of area	"Participants in rural areas were significantly less likely to have had an arsenic test" - Chappells, 2015
	Length of time in home	"The longer a respondent has lived in their home the less likely he/she is to have tested their well at all in the last 5 years" - Flanagan, 2015
	Live alone	"Having tested is significantly and positively associated with respondent's education and household income and negatively associated with living alone" - Flanagan, 2016a
	Knowledge about water quality hazards	Approximately 12% of respondents who had tested their well reported doing so because "water quality can change from time to time" - Malecki, 2017, Figure 3
	Knowledge about testing (Lack of)	Respondents that strongly disagreed or disagreed that they "have knowledge" of testing had lower odds of annual testing (OR: 0.21, 95%CI: 0.14-0.34)...Respondents who knew how often to test were more likely to test their water at least annually" - Kreutzwiser, 2011, Table 3
	Well age (Younger)	Having a well less than 10 years old increased odds of annual testing by a factor of 5 (OR: 5.0, 95%CI: 2.26, 11.06) - Kreutzwiser, 2011, Table 2
	Homeowner status (Renter)	There is a statistically significant difference in well testing prevalence between homeowners and renters (Previous testing = 42% in homeowners; 8% in renters; $\chi^2=23$, df=1, p-value<0.0001) - Postma, 2011, Table 3

Health Belief Model Construct		Factor Description	Factor Example
Individual Beliefs	Perceived Susceptibility	Do not drink from well	"Five respondents mentioned that they avoided drinking their water because of its poor quality, yet they still used it to bathe and wash clothes or dishes. Three interviewees saw avoiding water ingestion as a rationale for forgoing testing." - Fizer, 2018
		Water treatment system in use	Approximately 21% of respondents who do not test their well reported not doing so because "we treat and/or filter our water, so water testing is not needed" - Malecki, 2017, Figure 3
		Previous normal test result	No prior contamination was statistically significant with reducing odds of well testing (OR 0.37, 95%CI 0.24-0.58) - Kreutzwiser, 2011, Table 2
		Lack of perceived personal vulnerability	"While households generally agree with the severity of water and As-related health risks, they feel low personal vulnerability" - Flanagan, 2015
		Confidence in well water quality	Approximately 46% of respondents who do not test their well reported not doing so because "our water is probably fine" - Malecki, 2017, Figure 3
		No identified adverse health effects	Well Owner 21 Interview: "….it's a good well, there's nowhere that there's runoff, there's no reason that I could think that it would be contaminated. ***None of us are sick*** . So…maybe someday [I'll test]." - Imgrund, 2011
		Well reliability over time	Approximately 66% of respondents who do not test their well reported not doing so because they "have been drinking well water for years without any problem" - Malecki, 2017, Figure 3
	Perceived Severity	Lack of concern	"The most commonly cited reasons for not testing at all were a lack of concern and inconvenience...60% of well users reported having 'no' or 'low' concern about the health risks of arsenic in their well water" - Chappells, 2015
		Vulnerable groups in home	"Crude rates of testing in the last ten years versus not testing trended higher for...those with more children in the home." - Malecki, 2017
		Potential health risks of well water perceived as severe	"Perceptions of arsenic risk severity are stronger among those with a bachelor's degree than without" and education was associated with well testing behavior in this study - Flanagan, 2016c
	Perceived Benefits	Satisfy personal curiosity	6% of respondents reported their last well test was due to "curiosity" - Kreutzwiser, 2011
		Favorable attitude toward well testing	"All 13 well owners had positive attitudes towards sampling. In each instance, a positive attitude led directly to the intention and action of testing regularly...all suggested that the inconvenience [of testing] was minor or that testing was important enough to ignore, or to cope with, the inconvenience." - Imgrund, 2011
		Peace of mind	Respondents who reported that they previously tested for "peace of mind" had higher odds of annual testing (OR: 4.84, 95%CI: 2.54-9.25) - Kreutzwiser, 2011, Table 2
		Inform treatment options	Approximately 5% of respondents who have tested their well report doing so because a "water tested [was] needed to inform a decision about treating our well." - Malecki, 2017, Figure 3

**Individual Beliefs,** *continued*	**Perceived Barriers**	Property values may decrease or government interference for a positive test result	"A few participants also reported not testing their water because of fear of the "government's" response to a positive result: "*Once you put your water in to be tested, then they expect you to fix it if it's broken*"." - Jones, 2005
		Do not want to know about problems	Approximately 5% of respondents who do not test their well reported not doing so because they "do not want to know if there is a problem with my well water" - Malecki, 2017, Figure 3
		Perceived cost of test	"...the offer of a free test was commonly reported as a prompt for one-off testing." - Chappells, 2015
		Perceived inconvenience of test	"In response to an open-ended question, respondents explained why they did not test their private water supply or test it more frequently...with the most common explanation relating to the inconvenience of the testing process." Chappells, 2015
		Distrust of laboratory or organization performing test	Less than 1% of respondents reported not testing their well due to a "distrust [of] laboratory" - Jones, 2006
	**Perceived Self-Efficacy**	Belief that testing is easy	There was a lower perceived difficulty for testing among residents living in high-intervention towns - Flanagan, 2016b
		Cannot afford to fix problem	Approximately 8% of respondents who do not test their well reported not doing so because "a well water quality problem would be too expensive to fix" - Malecki, 2017, Figure 3
		Belief that they are capable of testing	"Confidence in ability to manage regularly testing and knowing who to contact for a well test are both significantly predicted by income", and income is positively associated with well testing behavior in this study. - Flanagan, 2016c
		Water quality is not in my control	Approximately 5% of respondents who do not test their well reported not doing so because "contamination is a result of urban growth/land use and not in my control" - Malecki, 2017, Figure 3
**Health Belief Model Construct**		**Factor Description**	**Factor Example**
**Cues to Action**		Policy promotes well testing	"During the first four and a half years of the New Jersey program [with enforcement mechanisms], 51,028 test results were submitted. These represented 13% of the estimated 400,000 private wells in New Jersey. By comparison, in the 20 years since enactment of the Oregon law, the total number of records submitted to the Oregon RET program [without enforcement mechanisms] represents only about 5% of the estimated 350,000 private wells in Oregon. This difference is likely the result of compliance problems with the Oregon PWT- RET law." - Hoppe, 2011
		Policy requires well testing	"86% of post-PWTA [New Jersey real-estate policy] respondents had their well tested at point of home purchase; this is statistically significantly different from pre-PWTA respondents at the 0.01 level." - Flanagan, 2016a
		Previous testing experience as routine	Respondents who reported that they previously tested for a "routine" had higher odds of annual testing (OR: 11.70, 95%CI: 6.04-22.68) - Kreutzwiser, 2011, Table 2
		Unknown water quality problem observed or suspected	Respondents who reported that they previously tested for a "suspected/noticed problem" had higher odds of annual testing (OR: 3,31, 95%CI: 1.55-7.06) - Kreutzwiser, 2011, Table 2

**Cues to Action,** *continued*	Heath problems observed	In describing what would prompt them to test their well, 36% said they would do so if they experienced “unexplained health problems such as frequent diarrhea or stomachaches” - Flanagan, 2015
	Exposure to testing information	"...we observed that well owners that were exposed to both MMC [mass-media campaign] and CBI [community-based intervention] were almost five times more likely to proceed with As [arsenic] screening than the non-exposed respondents when adjusting for age and gender ($p < 0.001$; Table 6)." - Renaud, 2011
	Received reminder	When asked what methods would help them increase the frequency of their well testing, approximately 50% responded "reminder mail-outs (flyers/brochures)" - Jones, 2006, Table 8
	Well testing program available	Approximately 14% of respondents who had tested their well reported doing so because "a well test program was offered in area" - Malecki, 2017, Figure 3
	Discounted (or free) well test available	In describing what would prompt them to test their well, 59% said they would do so if "well testing was available for free” - Flanagan, 2015
	Acquaintance/neighbor tested well	The second most commonly cited situation that would prompt a well test was "learning that their neighbors have contaminated water (74%)" - Flanagan, 2016a
	Change in aesthetics (smell, taste, color)	"Many interviewees reported that key prompts for testing their water were sensory or aesthetic problems…" - Chappells, 2015
	Well construction or maintenance	Approximately 11% of respondents who have tested their well report doing so because a "new well was constructed" and approximately 10% report doing so because "testing was needed after our well was repaired" - Malecki, 2017, Figure 3
	Real estate transaction, not required	"The top 5 reasons that homeowners reported having their well tested were….real estate transaction (30%)…." - Flanagan, 2015
	Learning of contaminated wells in the area	40% of respondents reported "Learning that some wells in their town have contaminated water" would prompt a well test - Flanagan, 2016a
	Occurrence of natural disaster	"A small number of respondents (<2%) selected “other--specify,” and identified several other motivations for testing including...occurrence of a natural or man-made disaster" - Malecki, 2017
	Promotional offer from a private company	Approximately 6% of respondents who have tested their well report doing so because of a "promotional offer from a private company" - Malecki, 2017, Figure 3
