# Supplementary material for: Risk Communication and Factors Influencing Private Well Testing Behavior: A Systematic Scoping Review
Source: Int J Environ Res Public Health. 2019 Nov 7;16(22):4333. doi: 10.3390/ijerph16224333 (PMC6888409; doi:10.3390/ijerph16224333)
Supplement: Supplementary file 1 [file ijerph-16-04333-s001.zip › Table S3_Summary of extracted data.pdf]

Table S3: Summary of data extracted from included studies

Title	Author(s), Publication Year	Factor categories addressed in article
Understanding the translation of scientific knowledge about arsenic risk exposure among private well water users in Nova Scotia	Chappells, et al., 2015	**Modifying Factors** (age, knowledge about testing; rurality of area, living in area of known hazards);<br><br>**Individual Beliefs** (lack of concern, vulnerable groups in home, perceived cost, perceived inconvenience) lack of perceived vulnerability);<br><br>**Cues to Action** (change in aesthetics, real estate transaction, policy requires well testing, exposure to testing information)
Barriers to managing private wells and septic systems in underserved communities: Mental models of homeowner decision making	Fizer, et al., 2018	**Modifying Factors** (knowledge about testing, knowledge about water quality hazards);<br><br>**Individual Beliefs** (perceived cost of test, lack of concern, do not drink from well)<br><br>**Cues to Action** (change in aesthetics)
Influences on domestic well water testing behavior in a Central Maine area with frequent groundwater arsenic occurrence	Flanagan, et al., 2015	**Modifying Factors** (age, education, length of time in home, living in area of known hazards)<br><br>**Individual Beliefs** (perceived cost of test, peace of mind, lack of perceived personal vulnerability)<br><br>**Cues to Action** (change in aesthetics, well construction or maintenance, real estate transaction, health problems observed, discounted well test available, acquaintance/neighbor tested well)
Arsenic in Private Well Water Part 1: Impact of the New Jersey Private Well Testing Act on household testing and mitigation behaviors	Flanagan, et al., 2016a	**Modifying Factors** (age, education, income, length of time in home, live alone)<br><br>**Individual Beliefs** (vulnerable groups in home)<br><br>**Cues to Action** (policy requires well testing, acquaintance/neighbor tested well, change in aesthetics, real estate transaction, learning of contaminated wells in area, discounted well test available)

Arsenic in private well water Part 2: Who benefits the most from traditional testing promotion?	Flanagan, et al., 2016b	**Modifying Factors** (age, education, income, length of time in home, live alone)<br><br>**Individual Beliefs** (vulnerable groups in home, perceived cost of test, belief that testing is easy)<br><br>**Cues to Action** (exposure to testing information)
Arsenic in private well water Part 3: Socioeconomic vulnerability to exposure in Maine and New Jersey	Flanagan, et al., 2016c	**Modifying Factors** (education level, income, knowledge about water quality hazards)<br><br>**Individual Beliefs** (potential health risks perceived as severe, favorable attitude toward well testing, belief that they are capable of testing, peace of mind)<br><br>**Cues to Action** (learning of contaminated wells in the area)
An investigation of bacteriological and chemical water quality and the barriers to private well water sampling in a Southwestern Ontario Community	Hexemer, et al., 2008	**Individual Beliefs** (perceived cost of test, perceived inconvenience of test)
Private well testing in Oregon from real estate transactions: an innovative approach toward a state-based surveillance system.	Hoppe, et al., 2011	**Cues to Action** (policy promotes well testing)
Influences on the water testing behaviors of private well owners	Imgrund, et al., 2011	**Modifying Factors** (knowledge about testing)<br><br>**Individual Beliefs** (confidence in well water quality, favorable attitude toward well testing, lack of concern, no identified adverse health effects, perceived inconvenience of test, peace of mind, well reliability over time)<br><br>**Cues to Action** (change in aesthetics, policy promotes well testing, real estate transaction, learning of contaminated wells in area, previous testing experience as routine, health problems observed, exposure to testing information, discounted well test available)

Public perception of drinking water from private water supplies: focus group analyses	Jones, et al., 2005	**Modifying Factors** (knowledge about testing)<br><br>**Individual Beliefs** (previous normal test result, confidence in well water quality, lack of concern, property values may decrease or government interference for positive test result, perceived inconvenience of test, cannot afford to fix problem)<br><br>**Cues to Action** (acquaintance/neighbor tested well, change in aesthetics, learning of contaminated wells in area, previous testing experience as routine, received reminder)
Public perceptions of drinking water: a postal survey of residents with private water supplies	Jones, et al., 2006	**Modifying Factors** (knowledge about testing)<br><br>**Individual Beliefs** (do not drink from well, water treatment system in use, previous normal test result, no identified adverse health effects, well reliability over time, lack of concern, perceived cost of test, perceived inconvenience of test, distrust of laboratory or organization performing test)<br><br>**Cues to Action** (exposure to testing information, received reminder, acquaintance/neighbor tested well)
Understanding stewardship behavior: Factors facilitating and constraining private water well stewardship	Kreutzwiser, et al., 2011	**Modifying Factors** (knowledge about testing)<br><br>**Individual Beliefs** (water treatment system in use, well age, previous normal test result, satisfy personal curiosity, peace of mind, perceived inconvenience of test)<br><br>**Cues to Action** (well construction or maintenance, policy requires well testing, previous testing experience as routine)

Groundwater nitrate contamination costs: A survey of private well owners	Lewandowski, et al., 2008	**Modifying Factors** (knowledge about testing)<br><br>**Individual Beliefs** (confidence in well water quality, perceived cost of test, lack of concern, perceived inconvenience of test)
Private-well stewardship among a general population based sample of private well-owners	Malecki, et al., 2017	**Modifying Factors** (gender, income, knowledge about testing, knowledge about water quality hazards, smoking status)<br><br>**Individual Beliefs** (do not want to know about problems, inform treatment options, confidence in well water quality, perceived cost of test, no identified adverse health effects, do not drink from well, water treatment system in use, well reliability over time, vulnerable groups in home, peace of mind, cannot afford to fix problem, water quality is not in my control)<br><br>**Cues to Action** (change in aesthetics, well construction or maintenance, real estate transaction, learning of contaminated wells in area, occurrence of natural disaster, promotional offer from private company, previous testing experience as routine, unknown water quality problem observed or suspected, well testing program available)
A Community-Driven Intervention in Tuftonboro, New Hampshire, Succeeds in Altering Water Testing Behavior	Paul, et al., 2015	*No factors*
Rural children's exposure to well water contaminants: Implications in light of the American Academy of Pediatrics' recent policy statement	Postma, et al., 2011	**Modifying Factors** (age, education, homeowner status, income)
Evaluation of the effectiveness of arsenic screening promotion in private wells: a quasi-experimental study	Renaud, et al., 2011	**Cues to Action** (previous testing experience as routine, exposure to testing information, acquaintance/neighbor tested well)

Application of a Modified Health Belief Model to the Pro-Environmental Behavior of Private Well Water Testing	Straub, et al., 2014	**Modifying Factors** (education, income)<br><br>**Individual Beliefs** (property values may decrease or government interference for positive result)<br><br>**Cues to Action** (received reminder)<br><br>*Many aspects of the HBM were also discussed, but were not extracted due to being included in broader HBM categories rather than specific factors and positive/negative associations. These broader categories and the survey questions that inform them are insightful for future research using the HBM in the context of well testing beahvior.<br><br>Statistically significant HBM categories of the that drove past well owner behavior included: "behavioral evaluation" (i.e. perceived barriers and perceived benefits) and socioeconomic status (i.e. modifying factors). Statistically significant aspects of the HBM that influenced future intentions to test included reminder cues to action. Not statistically significant categories related to past behavior or future intentions to test included threat perception, self-efficacy, other modifying factors, and water quality cue to action
